# 1-(2,3-Dimeth­oxy­benzyl­idene)-2-(2,4-dinitro­phen­yl)hydrazine

**DOI:** 10.1107/S1600536811013894

**Published:** 2011-04-16

**Authors:** Xianrong Xin, Min Li, Zhimin Chen, Ruitao Zhu

**Affiliations:** a Department of Chemistry and Chemical Engineering, Taiyuan Institute of Technology, Taiyuan 030008, People’s Republic of China; b Department of Chemistry, Taiyuan Normal University, Taiyuan 030031, People’s Republic of China

## Abstract

In the title compound, C_15_H_14_N_4_O_6_, the dihedral angle between the aromatic rings is 3.7 (4)°. The nitro groups make dihedral angles of 6.0 (4) and 5.2 (4)° with the parent ring and are oriented at 6.0 (6)° with respect to each other. The meth­oxy groups are inclined at 54.0 (2) and 2.5 (3)° with respect to the benzene ring to which they are attached. In the crystal, mol­ecules are linked by weak C—H⋯O inter­actions. The mol­ecular conformation is consolidated by an intra­molecular N—H⋯O hydrogen bond.

## Related literature

For general background to the properties of Schiff base compounds, see: Mufakkar *et al.* (2010 ▶); Tahir *et al.* (2010 ▶). For related structures, see: Salhin *et al.* (2007 ▶); Tameem *et al.* (2008 ▶); Shao *et al.* (2008 ▶).
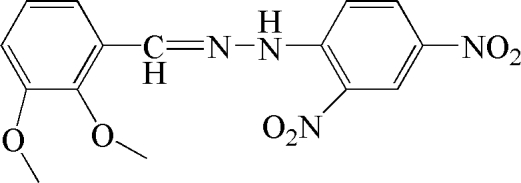


## Experimental

### 

#### Crystal data

C_15_H_14_N_4_O_6_
*M* *_r_* = 346.30
Triclinic, 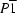

*a* = 7.8409 (8) Å
*b* = 7.9200 (9) Å
*c* = 13.8961 (14) Å
α = 85.038 (2)°
β = 82.773 (1)°
γ = 65.894 (1)°
*V* = 780.85 (14) Å^3^
*Z* = 2
Mo *K*α radiation
μ = 0.12 mm^−1^
*T* = 298 K
0.43 × 0.38 × 0.37 mm

#### Data collection

Bruker SMART CCD diffractometer
Absorption correction: multi-scan (*SADABS*; Bruker, 2007 ▶) *T*_min_ = 0.952, *T*_max_ = 0.958
4130 measured reflections
2725 independent reflections
1414 reflections with *I* > 2σ(*I*)
*R*_int_ = 0.032

#### Refinement

*R*[*F* ^2^ > 2σ(*F* ^2^)] = 0.063
*wR*(*F* ^2^) = 0.178
*S* = 1.06
2725 reflections
229 parameters
H-atom parameters constrained
Δρ_max_ = 0.28 e Å^−3^
Δρ_min_ = −0.25 e Å^−3^

### 

Data collection: *SMART* (Bruker, 2007 ▶); cell refinement: *SAINT* (Bruker, 2007 ▶); data reduction: *SAINT*; program(s) used to solve structure: *SHELXS97* (Sheldrick, 2008 ▶); program(s) used to refine structure: *SHELXL97* (Sheldrick, 2008 ▶); molecular graphics: *SHELXTL* (Sheldrick, 2008 ▶) and *PLATON* (Spek, 2009 ▶); software used to prepare material for publication: *SHELXTL*.

## Supplementary Material

Crystal structure: contains datablocks I, global. DOI: 10.1107/S1600536811013894/pv2405sup1.cif

Structure factors: contains datablocks I. DOI: 10.1107/S1600536811013894/pv2405Isup2.hkl

Additional supplementary materials:  crystallographic information; 3D view; checkCIF report

## Figures and Tables

**Table 1:** Hydrogen-bond geometry (Å, °)

*D*—H⋯*A*	*D*—H	H⋯*A*	*D*⋯*A*	*D*—H⋯*A*
N1—H1⋯O1	0.86	1.99	2.625 (4)	130
C14—H14*A*⋯O4^i^	0.96	2.48	3.431 (4)	170

Symmetry code: (i) 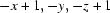.

**Fig. 1.**
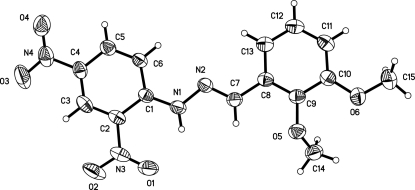
A view of the molecular structure of the title compound; displacement ellipsoids are drawn at the 30% probability level.

**Fig. 2.**
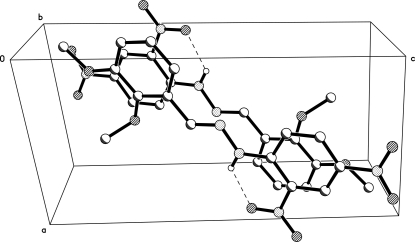
Part of the crystal structure of (I) with hydrogen bonds drawn as dashed lines.

## supplementary crystallographic information

### Comment

In view of the importance of hydrazone derivatives in chemical and biological applications (Shao *et al.*, 2008), a series of hydrazone derivatives has been prepared, and several X-ray structures have been reported (Salhin *et al.*, 2007; Aameem *et al.*, 2008). Here, we report the crystal structure of the title compound.

The bond distances and bond angles in the title compound (Fig. 1) are in agreement with the corresponding bond distances and angles reported in the crystale structures of closely related compounds, (Salhin *et al.*, 2007; Tameem *et al.*, 2008). In the crystal structure (Fig. 2), the molecules are linked by C—H···O weak interactions (Table 1). The molecular conformation is consolidated by an intramolecular N—H···O hydrogen bonding interaction (Table 1).

### Experimental

Equimolar quantities of 2,4-dinitrophenylhydrazine (0.99 g, 5.0 mmol) and 2,3-dimethoxybenzaldehyde (0.83 g, 5.0 mmol) were refluxed in ethanol (20 ml) for 30 min and rotary evaporated. The crystals of the title compound were growm by recrystallization from an ethanol solution.

### Refinement

H atoms were placed in idealized positions and allowed to ride on their respective parent atoms, with C—H = 0.93 and 0.96 Å, for methylene and ary H-atoms, respectively, and N—H = 0.86 Å with *U*_iso_(H)= 1.2*U*_eq_(methylene C/N) and 1.5*U*_eq_(aryl C).

### Crystal data

**Table:**

C_15_H_14_N_4_O_6_	*Z* = 2
*M**_r_* = 346.30	*F*(000) = 360
Triclinic, *P*1	*D*_x_ = 1.473 Mg m^−^^3^
Hall symbol: -P 1	Mo *K*α radiation, λ = 0.71073 Å
*a* = 7.8409 (8) Å	Cell parameters from 855 reflections
*b* = 7.9200 (9) Å	θ = 2.8–22.8°
*c* = 13.8961 (14) Å	µ = 0.12 mm^−^^1^
α = 85.038 (2)°	*T* = 298 K
β = 82.773 (1)°	Block, orange
γ = 65.894 (1)°	0.43 × 0.38 × 0.37 mm
*V* = 780.85 (14) Å^3^	

### Data collection

**Table:**

Bruker SMART CCD diffractometer	2725 independent reflections
Radiation source: fine-focus sealed tube	1414 reflections with *I* > 2σ(*I*)
graphite	*R*_int_ = 0.032
φ and ω scans	θ_max_ = 25.0°, θ_min_ = 2.8°
Absorption correction: multi-scan (*SADABS*; Bruker, 2007)	*h* = −9→9
*T*_min_ = 0.952, *T*_max_ = 0.958	*k* = −9→9
4130 measured reflections	*l* = −16→8

### Refinement

**Table:**

Refinement on *F*^2^	Secondary atom site location: difference Fourier map
Least-squares matrix: full	Hydrogen site location: inferred from neighbouring sites
*R*[*F*^2^ > 2σ(*F*^2^)] = 0.063	H-atom parameters constrained
*wR*(*F*^2^) = 0.178	*w* = 1/[σ^2^(*F*_o_^2^) + (0.0737*P*)^2^] where *P* = (*F*_o_^2^ + 2*F*_c_^2^)/3
*S* = 1.06	(Δ/σ)_max_ < 0.001
2725 reflections	Δρ_max_ = 0.28 e Å^−^^3^
229 parameters	Δρ_min_ = −0.25 e Å^−^^3^
0 restraints	Extinction correction: *SHELXL97* (Sheldrick, 2008), Fc^*^=kFc[1+0.001xFc^2^λ^3^/sin(2θ)]^-1/4^
Primary atom site location: structure-invariant direct methods	Extinction coefficient: 0.028 (5)

### Special details

**Table:**

Geometry. All e.s.d.'s (except the e.s.d. in the dihedral angle between two l.s. planes) are estimated using the full covariance matrix. The cell e.s.d.'s are taken into account individually in the estimation of e.s.d.'s in distances, angles and torsion angles; correlations between e.s.d.'s in cell parameters are only used when they are defined by crystal symmetry. An approximate (isotropic) treatment of cell e.s.d.'s is used for estimating e.s.d.'s involving l.s. planes.
Refinement. Refinement of *F*^2^ against ALL reflections. The weighted *R*-factor *wR* and goodness of fit *S* are based on *F*^2^, conventional *R*-factors *R* are based on *F*, with *F* set to zero for negative *F*^2^. The threshold expression of *F*^2^ > σ(*F*^2^) is used only for calculating *R*-factors(gt) *etc*. and is not relevant to the choice of reflections for refinement. *R*-factors based on *F*^2^ are statistically about twice as large as those based on *F*, and *R*- factors based on ALL data will be even larger.

### Fractional atomic coordinates and isotropic or equivalent isotropic displacement parameters (Å^2^)

**Table:**

	*x*	*y*	*z*	*U*_iso_*/*U*_eq_	
N1	0.6288 (3)	0.1887 (3)	0.55122 (17)	0.0672 (7)	
H1	0.7290	0.1995	0.5243	0.081*	
N2	0.4685 (3)	0.2598 (3)	0.50406 (17)	0.0627 (6)	
N3	0.9691 (3)	0.0341 (4)	0.6504 (3)	0.0789 (8)	
N4	0.6248 (5)	−0.1660 (4)	0.9164 (2)	0.0889 (9)	
O1	0.9777 (3)	0.1238 (4)	0.57412 (19)	0.0936 (8)	
O2	1.1061 (3)	−0.0474 (4)	0.6963 (2)	0.1191 (10)	
O3	0.7741 (4)	−0.2406 (4)	0.9539 (2)	0.1259 (11)	
O4	0.4763 (4)	−0.1674 (4)	0.95499 (18)	0.1107 (9)	
O5	0.5156 (3)	0.5336 (3)	0.25258 (14)	0.0734 (6)	
O6	0.2347 (3)	0.6845 (3)	0.13346 (16)	0.0810 (7)	
C1	0.6302 (3)	0.1025 (3)	0.6391 (2)	0.0549 (7)	
C2	0.7926 (3)	0.0256 (4)	0.6903 (2)	0.0626 (8)	
C3	0.7883 (4)	−0.0608 (4)	0.7802 (2)	0.0681 (8)	
H3	0.8956	−0.1094	0.8130	0.082*	
C4	0.6287 (4)	−0.0754 (4)	0.8214 (2)	0.0652 (8)	
C5	0.4661 (4)	−0.0033 (4)	0.7723 (2)	0.0651 (8)	
H5	0.3567	−0.0136	0.8008	0.078*	
C6	0.4688 (4)	0.0809 (4)	0.6841 (2)	0.0609 (7)	
H6	0.3609	0.1260	0.6518	0.073*	
C7	0.4850 (4)	0.3361 (4)	0.4206 (2)	0.0627 (7)	
H7	0.6000	0.3394	0.3968	0.075*	
C8	0.3275 (4)	0.4181 (4)	0.3620 (2)	0.0588 (7)	
C9	0.3505 (4)	0.5101 (4)	0.2741 (2)	0.0602 (7)	
C10	0.2012 (4)	0.5909 (4)	0.2156 (2)	0.0641 (8)	
C11	0.0346 (4)	0.5735 (4)	0.2461 (2)	0.0744 (9)	
H11	−0.0643	0.6240	0.2074	0.089*	
C12	0.0117 (4)	0.4825 (4)	0.3330 (3)	0.0756 (9)	
H12	−0.1026	0.4740	0.3525	0.091*	
C13	0.1564 (4)	0.4046 (4)	0.3907 (2)	0.0684 (8)	
H13	0.1404	0.3428	0.4488	0.082*	
C14	0.6191 (4)	0.4797 (5)	0.1608 (2)	0.0847 (10)	
H14A	0.5771	0.3991	0.1323	0.127*	
H14B	0.7503	0.4159	0.1691	0.127*	
H14C	0.5998	0.5877	0.1190	0.127*	
C15	0.0951 (5)	0.7525 (5)	0.0675 (3)	0.1016 (12)	
H15A	0.0718	0.6516	0.0472	0.152*	
H15B	0.1378	0.8107	0.0118	0.152*	
H15C	−0.0187	0.8412	0.0987	0.152*	

### Atomic displacement parameters (Å^2^)

**Table:**

	*U*^11^	*U*^22^	*U*^33^	*U*^12^	*U*^13^	*U*^23^
N1	0.0509 (13)	0.0903 (17)	0.0649 (17)	−0.0324 (12)	−0.0047 (11)	−0.0079 (13)
N2	0.0547 (14)	0.0696 (15)	0.0639 (16)	−0.0245 (12)	−0.0081 (11)	−0.0030 (12)
N3	0.0472 (15)	0.0818 (19)	0.110 (2)	−0.0237 (14)	−0.0100 (15)	−0.0246 (16)
N4	0.117 (3)	0.088 (2)	0.081 (2)	−0.0550 (19)	−0.0386 (19)	0.0088 (16)
O1	0.0661 (14)	0.133 (2)	0.0922 (18)	−0.0513 (14)	0.0068 (12)	−0.0250 (15)
O2	0.0526 (13)	0.124 (2)	0.180 (3)	−0.0300 (13)	−0.0379 (16)	0.0068 (18)
O3	0.136 (2)	0.138 (2)	0.114 (2)	−0.0557 (18)	−0.0725 (19)	0.0353 (17)
O4	0.142 (2)	0.140 (2)	0.0873 (19)	−0.095 (2)	−0.0317 (16)	0.0251 (15)
O5	0.0724 (13)	0.0967 (15)	0.0642 (13)	−0.0473 (12)	−0.0049 (10)	−0.0064 (10)
O6	0.0827 (14)	0.0920 (15)	0.0730 (15)	−0.0389 (12)	−0.0219 (11)	0.0126 (12)
C1	0.0492 (15)	0.0540 (15)	0.0625 (19)	−0.0194 (13)	−0.0076 (12)	−0.0111 (13)
C2	0.0461 (16)	0.0640 (18)	0.080 (2)	−0.0201 (13)	−0.0103 (14)	−0.0183 (16)
C3	0.0621 (19)	0.0584 (17)	0.087 (2)	−0.0196 (15)	−0.0281 (16)	−0.0119 (16)
C4	0.077 (2)	0.0615 (17)	0.066 (2)	−0.0324 (15)	−0.0250 (15)	−0.0003 (14)
C5	0.0628 (18)	0.0648 (18)	0.076 (2)	−0.0329 (15)	−0.0142 (15)	0.0020 (15)
C6	0.0515 (16)	0.0651 (17)	0.070 (2)	−0.0256 (13)	−0.0144 (13)	−0.0007 (15)
C7	0.0573 (17)	0.0741 (19)	0.0600 (19)	−0.0302 (15)	−0.0019 (13)	−0.0060 (15)
C8	0.0543 (16)	0.0630 (17)	0.0602 (18)	−0.0240 (14)	−0.0005 (13)	−0.0129 (13)
C9	0.0572 (17)	0.0653 (17)	0.0628 (19)	−0.0287 (14)	−0.0009 (13)	−0.0125 (14)
C10	0.0627 (18)	0.0639 (18)	0.067 (2)	−0.0250 (15)	−0.0085 (14)	−0.0074 (15)
C11	0.0620 (19)	0.075 (2)	0.089 (2)	−0.0260 (16)	−0.0185 (16)	−0.0074 (17)
C12	0.0578 (18)	0.078 (2)	0.095 (3)	−0.0327 (16)	0.0006 (17)	−0.0115 (18)
C13	0.0627 (18)	0.0689 (19)	0.076 (2)	−0.0293 (15)	−0.0014 (15)	−0.0098 (15)
C14	0.080 (2)	0.113 (3)	0.072 (2)	−0.051 (2)	−0.0011 (16)	−0.0033 (18)
C15	0.095 (3)	0.120 (3)	0.084 (3)	−0.035 (2)	−0.032 (2)	0.019 (2)

### Geometric parameters (Å, °)

**Table:**

N1—C1	1.345 (3)	C5—C6	1.347 (4)
N1—N2	1.375 (3)	C5—H5	0.9300
N1—H1	0.8600	C6—H6	0.9300
N2—C7	1.278 (3)	C7—C8	1.455 (4)
N3—O2	1.230 (3)	C7—H7	0.9300
N3—O1	1.234 (3)	C8—C13	1.395 (4)
N3—C2	1.451 (4)	C8—C9	1.397 (4)
N4—O4	1.223 (3)	C9—C10	1.408 (4)
N4—O3	1.236 (3)	C10—C11	1.379 (4)
N4—C4	1.450 (4)	C11—C12	1.382 (4)
O5—C9	1.375 (3)	C11—H11	0.9300
O5—C14	1.420 (3)	C12—C13	1.372 (4)
O6—C10	1.363 (3)	C12—H12	0.9300
O6—C15	1.420 (3)	C13—H13	0.9300
C1—C6	1.407 (4)	C14—H14A	0.9600
C1—C2	1.421 (4)	C14—H14B	0.9600
C2—C3	1.375 (4)	C14—H14C	0.9600
C3—C4	1.355 (4)	C15—H15A	0.9600
C3—H3	0.9300	C15—H15B	0.9600
C4—C5	1.404 (4)	C15—H15C	0.9600
			
C1—N1—N2	120.6 (2)	C8—C7—H7	119.4
C1—N1—H1	119.7	C13—C8—C9	119.6 (3)
N2—N1—H1	119.7	C13—C8—C7	121.7 (3)
C7—N2—N1	114.9 (2)	C9—C8—C7	118.7 (2)
O2—N3—O1	122.2 (3)	O5—C9—C8	117.6 (2)
O2—N3—C2	117.5 (3)	O5—C9—C10	121.8 (3)
O1—N3—C2	120.3 (3)	C8—C9—C10	120.2 (3)
O4—N4—O3	123.2 (3)	O6—C10—C11	125.7 (3)
O4—N4—C4	119.1 (3)	O6—C10—C9	115.8 (3)
O3—N4—C4	117.7 (3)	C11—C10—C9	118.4 (3)
C9—O5—C14	118.8 (2)	C10—C11—C12	121.4 (3)
C10—O6—C15	117.7 (2)	C10—C11—H11	119.3
N1—C1—C6	120.8 (2)	C12—C11—H11	119.3
N1—C1—C2	122.6 (3)	C13—C12—C11	120.4 (3)
C6—C1—C2	116.5 (3)	C13—C12—H12	119.8
C3—C2—C1	121.0 (3)	C11—C12—H12	119.8
C3—C2—N3	117.3 (3)	C12—C13—C8	120.0 (3)
C1—C2—N3	121.8 (3)	C12—C13—H13	120.0
C4—C3—C2	120.4 (3)	C8—C13—H13	120.0
C4—C3—H3	119.8	O5—C14—H14A	109.5
C2—C3—H3	119.8	O5—C14—H14B	109.5
C3—C4—C5	120.2 (3)	H14A—C14—H14B	109.5
C3—C4—N4	120.1 (3)	O5—C14—H14C	109.5
C5—C4—N4	119.6 (3)	H14A—C14—H14C	109.5
C6—C5—C4	119.9 (3)	H14B—C14—H14C	109.5
C6—C5—H5	120.0	O6—C15—H15A	109.5
C4—C5—H5	120.0	O6—C15—H15B	109.5
C5—C6—C1	121.9 (2)	H15A—C15—H15B	109.5
C5—C6—H6	119.0	O6—C15—H15C	109.5
C1—C6—H6	119.0	H15A—C15—H15C	109.5
N2—C7—C8	121.3 (3)	H15B—C15—H15C	109.5
N2—C7—H7	119.4		
			
C1—N1—N2—C7	−179.1 (2)	C2—C1—C6—C5	2.0 (4)
N2—N1—C1—C6	0.9 (4)	N1—N2—C7—C8	−179.8 (2)
N2—N1—C1—C2	179.5 (2)	N2—C7—C8—C13	−5.0 (4)
N1—C1—C2—C3	179.7 (2)	N2—C7—C8—C9	175.9 (2)
C6—C1—C2—C3	−1.7 (4)	C14—O5—C9—C8	129.0 (3)
N1—C1—C2—N3	−0.4 (4)	C14—O5—C9—C10	−57.3 (4)
C6—C1—C2—N3	178.2 (2)	C13—C8—C9—O5	174.7 (2)
O2—N3—C2—C3	4.9 (4)	C7—C8—C9—O5	−6.3 (4)
O1—N3—C2—C3	−173.6 (3)	C13—C8—C9—C10	0.9 (4)
O2—N3—C2—C1	−174.9 (3)	C7—C8—C9—C10	180.0 (2)
O1—N3—C2—C1	6.6 (4)	C15—O6—C10—C11	−7.7 (4)
C1—C2—C3—C4	0.6 (4)	C15—O6—C10—C9	173.9 (3)
N3—C2—C3—C4	−179.3 (2)	O5—C9—C10—O6	3.7 (4)
C2—C3—C4—C5	0.3 (4)	C8—C9—C10—O6	177.2 (2)
C2—C3—C4—N4	−179.8 (2)	O5—C9—C10—C11	−174.8 (2)
O4—N4—C4—C3	175.4 (3)	C8—C9—C10—C11	−1.4 (4)
O3—N4—C4—C3	−5.5 (4)	O6—C10—C11—C12	−177.0 (3)
O4—N4—C4—C5	−4.7 (4)	C9—C10—C11—C12	1.3 (4)
O3—N4—C4—C5	174.4 (3)	C10—C11—C12—C13	−0.9 (5)
C3—C4—C5—C6	0.0 (4)	C11—C12—C13—C8	0.4 (5)
N4—C4—C5—C6	−179.9 (2)	C9—C8—C13—C12	−0.5 (4)
C4—C5—C6—C1	−1.2 (4)	C7—C8—C13—C12	−179.5 (3)
N1—C1—C6—C5	−179.3 (2)		

### Hydrogen-bond geometry (Å, °)

**Table:**

*D*—H···*A*	*D*—H	H···*A*	*D*···*A*	*D*—H···*A*
N1—H1···O1	0.86	1.99	2.625 (4)	130
N1—H1···N3	0.86	2.60	2.913 (4)	103
C3—H3···O2	0.93	2.33	2.654 (4)	100
C6—H6···N2	0.93	2.44	2.766 (4)	100
C7—H7···O5	0.93	2.41	2.737 (4)	101
C14—H14A···O4^i^	0.96	2.48	3.431 (4)	170

Symmetry codes: (i) −*x*+1, −*y*, −*z*+1.
